# Impact of Cooking Preparation on *In Vitro* Digestion of Eggs Simulating Some Gastrointestinal Alterations in Elders

**DOI:** 10.1021/acs.jafc.0c07418

**Published:** 2021-04-09

**Authors:** Ever Hernández-Olivas, Sara Muñoz-Pina, Ana Andrés, Ana Heredia

**Affiliations:** Instituto Universitario de Ingeniería de Alimentos para el Desarrollo, Universitat Politècnica de València, Camino de Vera s/n, 46022 Valencia, Spain

**Keywords:** aging, egg, cooking methods, macronutrients digestibility, vitamin bioaccessibility

## Abstract

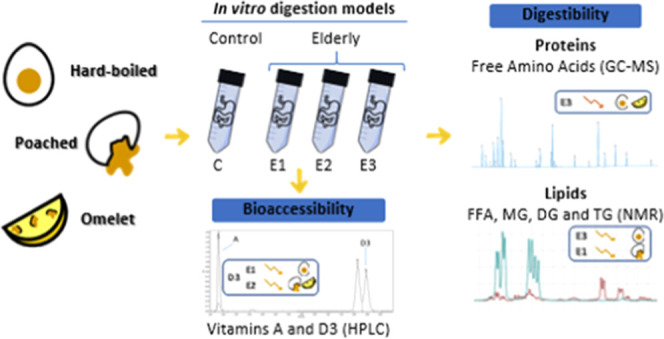

This study aimed to *in vitro* assess the impact of the cooking process of eggs (hard-boiled, poached, and omelet) on nutrients digestibility and vitamins A and D3 bioaccessibility under elderly gastrointestinal (GI) conditions. Three elderly digestion models were mimicked: oral (E1); oral and gastric (E2); and oral, gastric, and intestinal (E3), and a healthy adult model (C). Proteolysis extent reduced after digestion of omelet under the E3 model (*p* < 0.05) (up to 37% of reduction). Thus, hard-boiled and poached were more recommendable to enhance protein digestibility in elders. Altered GI conditions negatively influence neither the absorbable lipid fraction nor the cholesterol stability. Finally, vitamin A bioaccessibility was not affected but D3 slightly decreased with the elderly (E3). Hence, the digestion of nutrients was dependent on the resulting matrix, poached being the greater supplier of protein and lipid end-digestion products. Poached and omelet, however, offer a high net supply of bioaccessible vitamin D3 for elders.

## Introduction

Current prospects confirm that the population continues to considerably grow because of both high fertility and life expectancy. At the same time, it is expected that the number of people aged 65 years or over surpasses infants and youth in number by 2050.^1^ Consequently, elders wellness is a global concern that involves lifestyle and nutritional issues.^2^ The European Society for Clinical Nutrition and Metabolism recommends the elders to increase the consumption of rich-protein foods with high amounts of micronutrients,^3^ and especially those rich in essential amino acids such as leucine or tryptophan.^4^ Besides, healthy lipids, minerals, and vitamins are also important due to their relevance as immune modulators and their contribution to the bone health of these subjects.^5^ Physiological functions declining with aging include body composition, brain function, gastrointestinal (GI) tract function, fluid balance, bones and joints, or cardiovascular system, among others.^6^ Sarcopenia, loss of muscle mass associated with a protein deficit, asthenia, depression, or weakness of the immune system often occur in elderly.^7,8^ The masticatory deficiency in elderly, i.e., leading to food boluses with larger particle size distribution and more difficult to swallow, has been reported to influence the nutrients digestibility.^9^ Also, a decline in the GI tract function has been reported to be partially responsible for the protein deficit. The secretion of digestive fluids and enzymes, saliva, peristaltic contractions, and chyme passage rates could be suboptimal, resulting in maldigestion and malabsorption of nutrients, especially proteins and vitamins.^10−12^

Among the dietary protein and micronutrients sources, egg is considered as a moderate calorie source (about 140 kcal/100 g) and the lowest-cost animal source of proteins, vitamin A, iron, vitamin B12, riboflavin, and choline, as well as the second lowest-cost source for zinc and calcium. Egg proteins are distributed equally between egg white and egg yolk, while lipids, vitamins, and minerals are essentially concentrated in egg yolk.^13^ Raw egg yolk contains a high amount of vitamin A and D3 (371 and 5.4 μg/100 g, respectively), among others.^13^ Proteins provide a reasonable supply of amino acids of biological value,^14^ with a digestible indispensable amino acid score (DIAAS) value of 1.13 in the same high level of the whole milk with 1.14 score.^15^ The relative amount of mono- and polyunsaturated to saturated fatty acids in yolk is particularly higher than that in other animal-derived foods. Besides, even though egg cholesterol content is high, it has been reported to not negatively contribute to the increase in plasma total cholesterol.^13^ Therefore, a regular egg consumption of about 6 per week is advisable.^16^ Thus, egg is one of the most eaten food over the world and is served in such a variety of ways and recipes.^17^

Egg meal preparation often involves a heating treatment resulting in protein denaturation, greater vitamins, and minerals availability,^18^ as well as loss and antinutritional factors decrease, among others. The extent of these changes will depend on the way of cooking and the intensity of the heating.^14^ Additionally, cooking implies a series of structural changes, which could modulate digestion and absorption rates (i.e., amino acid isomerization and desulfurization, reactions with sugars and lipids, etc.), therefore having an impact on health benefits coming from egg consumption.^19^ Among the most common ways of cooking eggs, hard and soft boiled, hard and soft scrambled, omelet, sunny side up, etc. can be mentioned. The literature reports the impact of the egg protein structure on proteolysis in model systems consisting in white gels. Studies performed at static^19−21^ and dynamic *in vitro* systems^21,22^ clearly evidencing the role of the matrix structure. However, information related to the modulation of egg protein digestibility neither by cooking nor under elderly GI conditions has been previously reported in real foods.

In this context, this study aims at *in vitro* analyzing the impact of elderly gastrointestinal conditions and egg cooking (hard-boiled, poached, and omelet) on proteolysis, lipolysis, and vitamins A and D3 bioaccessibility.

## Materials and Methods

### Chemicals

Pepsin from the porcine gastric mucosa (3200–4500 U/mg), pancreatin (8 × USP) from porcine pancreas, bile bovine (dried, unfractionated), analytical-grade salts (potassium chloride, potassium dihydrogen phosphate, sodium bicarbonate, sodium chloride, magnesium chloride, ammonium carbonate, calcium chloride, and potassium sulfate), boric acid, hydrochloric acid (37%), sulfuric acid (95–97%), tetrahydrofuran (HPLC grade), methanol (HPLC grade), retinol (≥99%, HPLC grade), cholecalciferol (≥98%, HPLC grade), and sodium hydroxide were obtained from Sigma-Aldrich (Deisenhofen, Germany). Also, petroleum ether (VWR Chemicals), acetonitrile (HPLC grade, JT Baker), and EZ-Faast amino acid kit (Phenomenex) were used.

Standard eggs were purchased at local stores in Valencia (Spain).

### Sample Preparation

Fresh hen eggs were cooked according to Asensio-Grau et al.^23^ and immediately characterized or *in vitro* digested. For the hard-boiled whole shell, eggs were boiled with water covering the eggs for 10 min (95 ± 5 °C) and cooled under running tap water for 5 min, and they were immediately peeled. For poached preparation, eggs were broken and wrapped into cling-film before boiling them with boiling water for 4 min (95 ± 5 °C) and cooled under running tap water for 5 min. For omelet, a white/yolk ratio of 70:30 (w:w) was mixed and stirred for 1 min before microwave cooking at 12.5 W/g for 80 s without oil addition. The egg white and yolks resulted from hard-boiling and poaching were separated to be added to the digestion tubes in the same white:yolk ratio as in omelet.

### Compositional Analysis

After cooking, moisture, ashes, fat, and protein contents were determined using the official methods to be 934.01, 942.05, 920.39, and 960.52,^24^ respectively. Carbohydrates were calculated by difference (100 g minus the sum of grams of water, ashes, lipids, and protein, in wet basis).^25^ Besides, 5 g of samples was subjected to saponification and extraction of vitamins A (retinol) and D3 (cholecalciferol) according to the protocol published by Castaneda and Lee.^26^ Both liposoluble vitamins were separated by chromatography (RP-HPLC) and detected at 265 and 325 nm for vitamin D3 and vitamin A, respectively.^27^ Additionally, cold lipid extraction was performed to analyze the egg lipid profile by means of proton nuclear magnetic resonance (^1^H NMR) (Bruker, model 400/R), according to Nieva-Echevarría et al.^28^ The molar percentages of triglycerides (TG), diglycerides (1,2-DG and 1,3-DG), monoglycerides (1-MG and 2-MG), and free fatty acids (FFA) were determined in the samples. To assess its stability after the egg cooking and digestion, the cholesterol content was also quantified by ^1^H NMR, as a minor lipidic component.

Determinations were performed by triplicate in at least three independent eggs for each cooking method.

### Static *In Vitro* Simulation of GI Digestion

**Figure 1:**
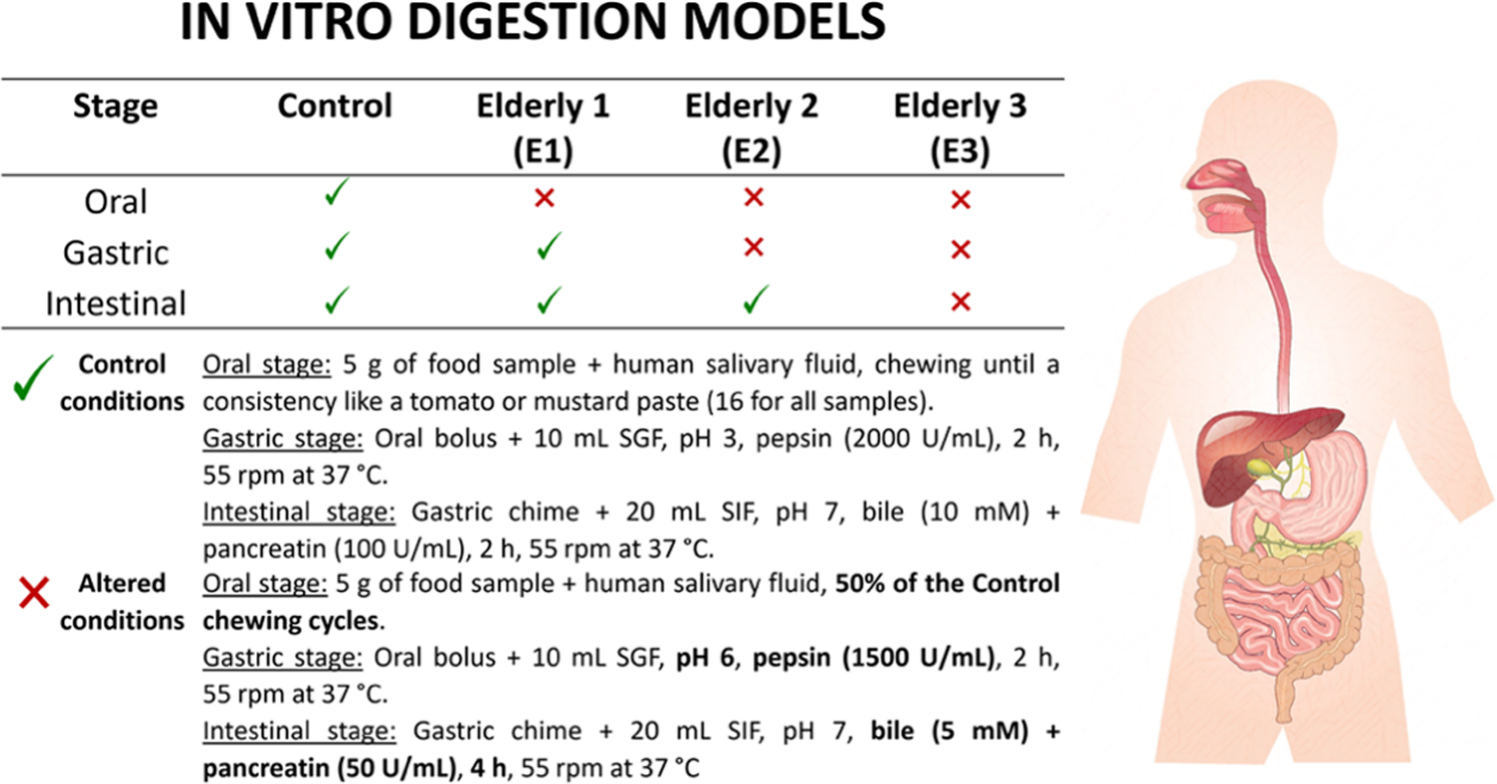
Specific gastrointestinal conditions set for the four *in vitro* digestion models of this study.

Four *in vitro* models were stated according to Hernández-Olivas et al.^27^ to determine the contribution of the different alterations and deterioration occurring with aging (i.e., mastication deficiency, secretion of digestive fluids and enzymes, saliva, GI tract contractions, and chyme passage rates)^9,12^ on the macronutrients digestibility and micronutrients bioaccessibility in the cooked eggs. Figure 1 gathers the specific conditions of each simulation model (Elderly 1 (E1), Elderly 2 (E2), Elderly 3 (E3), and control (C)). GI-altered conditions of elderly models E1, E2, and E3 were based on Shani-Levi et al.,^12^ while the C model corresponded to Minekus et al.^29^ Three independent digestion assays were carried out for each C, E1, E2, and E3 GI condition. Cooked eggs (5 g, hard-boiled, poached, and omelet) ensuring a 70:30 white/yolk ratio were digested by triplicate under each GI model (C, E1, E2, and E3). Gastric and intestinal stages were *in vitro* simulated, while oral stage was *in vivo* performed by a volunteer with healthy dentition. The number of mastication cycles to reach a bolus with similar physical characteristics to that of a tomato or mustard paste was established at 16.^30^ Once this parameter was established, chewing cycles were reduced to 50% to mimic suboptimal oral conditions given in elders.^27^ Before digestion experiments, gastric (SGF) and intestinal (SIF) digestion fluids were prepared fresh daily from stock solutions and the enzymatic activity of digestive enzymes was tested following the protocol proposed by Minekus et al.^29^

After *in vitro* digestion, sample pH was adjusted to 5 and kept in an ice bath for 10 min to inhibit the enzymatic reactions before fraction separation. Separation of the bioaccessible fraction (liquid phase) from the remaining solid phase was performed by centrifuging at 4000*g* for 5 min at 10 °C, and the supernatant was collected as a bioaccessible fraction. Aliquots of the bioaccessible fraction were immediately frozen and stored until their use for the analytical determinations.

### Analytical Determinations in Digesta

#### Free Amino Acids

Free amino acids (essential and nonessential amino acids (EAA and NEAA)) resulting from protein digestion were determined through the protocol published by Peinado et al.^31^ with some amendments. The resulted amino acids were classified into groups according to their chemical structure (hydrophobic amino acids (HAA = Ala, Val, Ile, Leu, Tyr, Phe, Trp, Pro, Met, Cys); positively charged amino acids (PCAA = Lys, His); negatively charged amino acids (NCAA = Asp, Asn, Glu, Gln); aromatic amino acids (AAA = Phe, Trp, Tyr); and sulfur-containing amino acids (SCAA = Cys, Met)).^32^ Briefly, 100 μL of bioaccessible fraction was derivatized using the EZ-Faast amino acid kit and analyzed using gas chromatography–mass spectrometry (GC–MS) (Agilent Technologies, Injector 7683B series, Network GC System 6890N series, Inert Mass Selective Detector 5975 series, MSD ChemStation software). Norvaline was used as internal standard. The extent of proteolysis based on free amino acids was calculated according to eq 1
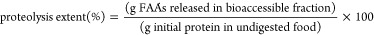
1where the FAA released corresponds to the sum of the free amino acids in the bioaccessible fraction.

#### Lipidic End-Digestion Products

Digesta samples were subjected to cold liquid–liquid extraction, and the composition of the lipid phase, including cholesterol, was determined by ^1^H NMR following the same procedure described in the Compositional Analysis section. Thus, absorbable and nonabsorbable lipid fractions, as well as the lipolysis extent, were calculated according to eqs 2–4
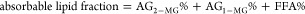
2
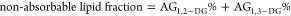
3
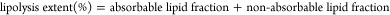
4where 1,2-DG and 1,3-DG correspond to diglycerides; 1-MG and 2-MG to monoglycerides; and FFA to free fatty acids obtained in the digested samples.

#### Vitamin A and D3 Bioaccessibility

Bioaccessible fraction (20 mL) was subjected to saponification and extraction to determine the bioaccessibility of vitamin A and D3 following the same protocol as for total vitamin content in undigested cooked eggs (Compositional Analysis section). Vitamin bioaccessibility was calculated according to eq 5
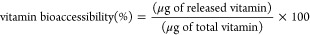
5where the amount of released vitamin represents the recovered part in the bioaccessible fraction after *in vitro* digestion and the total amount of vitamin found in the cooked eggs before *in vitro* digestion.

### Statistical Analysis

An analysis of variance (multivariate ANOVA) was performed and multiple-range test was determined by the less significant difference (LSD) of Fisher’s test to identify homogeneous groups between models and cooked eggs using Statgraphics Centurion XVII software with a confidence level of 95% (*p* < 0.05). Also, a principal component analysis (PCA) was applied to find the relationship among the experimental data (EAA/NEAA ratio, total, HAA, PCAA, NCAA, AAA, and SCAA proteolysis extents; absorbable and nonabsorbable lipid fractions; lipolysis extent; cholesterol content; and vitamin A and D3 bioaccessibility) obtained from *in vitro* digestion studies carried out in cooked eggs under elderly (E1, E2, and E3) or standard (C) GI conditions.

## Results and Discussion

### Effect of Cooking on Egg Composition

The nutritional composition of eggs was evaluated immediately after being cooked, and the values are presented in Table 1. Even though the egg nutritional contents are highly dependent on the hen feed composition,^33^ macronutrients content (protein and fat) were close to those reported for noncooked egg.^34−37^ Therefore, no losses of protein or fat were observed during cooking. Regarding the water content, the shell (in hard-boiled egg) and the plastic film used during poaching avoided the sample dehydration compared with the open-air preparation of omelet. Concerning the analyzed vitamins, cooked eggs presented lower values of vitamin A but similar to D3 compared to the contents reported in fresh egg.^34,35,37^ A decrease of yolk hydrophobic micronutrients has been previously reported after cooking,^38^ vitamin A being more sensitive to light, oxygen, and temperature than other liposoluble vitamins.^39^ In addition, Hemery et al.^40^ report a greater effect of photolysis than oxidation on vitamin A. Reasonably, the lower vitamin A content found in omelet, compared to hard-boiled and poached egg, can be due to a greater yolk exposure to light and oxygen than during the other cooking ways. In omelet preparation, the shell is removed and the yolk and egg white were mixed, stirred, and placed in a plate, resulting in a larger interphase surface to thermal heating than in boiled or poached. With respect to vitamin D3, omelet presented higher content than hard-boiled or poached eggs. Hemery et al.^40^ report that the impact of light or oxygen exposure on vitamin D3 is not as severe as for vitamin A. Vitamin D3 seems to be sensible to heat and decrease as long as the processing time increases.^41,42^ Thus, the lower cooking time involved in the microwave preparation of omelet (80 s compared to 4 and 10 min, respectively) could be associated with the better preservation of vitamin D3 compared to boiling and poaching.

**Table 1:** Total Contents (per 100 g Dry Basis) of Water, Protein, Fat, Ashes, Carbohydrates, Vitamin A and Vitamin D3 of Hard-boiled, Poached and Omelet Eggs^e^

nutrient content	raw^d^	hard-boiled	poached	omelet
water (g)	292–308	310 ± 3^b^	319 ± 3^c^	154 ± 2^a^
protein (g)	47–52	51.6 ± 0.2^b^	49.5 ± 0.6^a^	51.8 ± 0.3^b^
fat (g)	35–48	35.4 ± 0.6^a^	35.0 ± 1.0^a^	33.4 ± 1.7^a^
ashes (g)	3.4–3.6	5.9 ± 0.1^a^	5.9 ± 0.1^a^	5.8 ± 0.1^a^
carbohydrates (g)	0.7–3.8	4.9 ± 0.1^b^	4.6 ± 0.1^a^	5.1 ± 0.1^c^
vitamin A (μg)	560–1112	690 ± 30^b^	700 ± 30^b^	376 ± 18^a^
vitamin D3 (μg)	5–12	6.3 ± 0.3^a^	6.5 ± 0.3^a^	11.2 ± 0.4^b^

a,b,c Different lowercase letters indicate significant differences between foods, with a significance level of 95% (*p* < 0.05).

d Intervals based on literature.^34−37^

e Data shown are mean values and standard deviation from three independent eggs.

### Effect of Egg Cooking on Gastrointestinal Proteolysis in Elders

**Figure 2:**
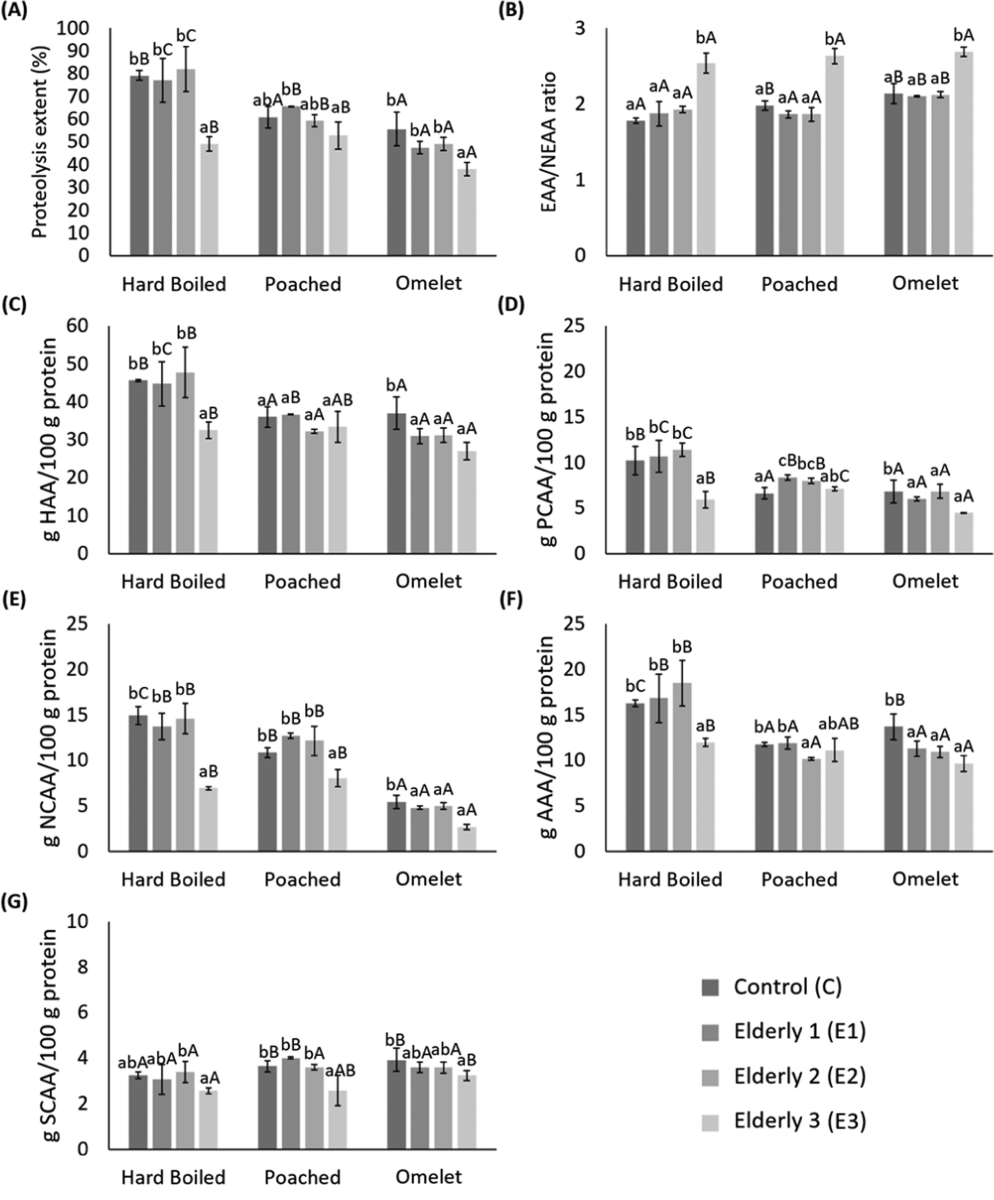
Proteolysis extent (%) (g FAAs released/100 g protein) (A); essential and nonessential amino acids ratio (EAA/NEAA ratio) (B); and amino acid quantities (g/100 g of protein) classified by chemical structure (HHA (C), PCAA (D), NCAA (E), AAA (F), and SCAA (G)) found in hard-boiled, poached, and omelet eggs *in vitro* digested under C (control), E1 (Elderly 1), E2 (Elderly 2), and E3 (Elderly 3) GI conditions. EAA = (Val, Leu, Ile, Thr, Met, Phe, Lys, His, Trp); NEAA = (Ala, Gly, Ser, Pro, Asn, Asp, Glu, Tyr, Cys). Hydrophobic amino acids (HAA = Ala, Val, Ile, Leu, Tyr, Phe, Trp, Pro, Met, Cys); positively charged amino acids (PCAA = Lys, His); negatively charged amino acids (NCAA = Asp, Asn, Glu, Gln); aromatic amino acids (AAA = Phe, Trp, Tyr); and sulfur-containing amino acids (SCAA = Cys, Met). Data shown are mean values from triplicates and the standard deviation. Different lowercase letters indicate significant differences between models, and different capital letters indicate significant differences between cooking methods, with a significance level of 95% (*p* < 0.05).

Figure 2A shows the proteolysis extent (%) obtained from the free amino acid profile (Table 2) achieved after *in vitro* gastrointestinal digestion of boiled, poached, and omelet eggs simulating different models (standardized (C) and elderly (E1, E2, and E3)). It can be noted that proteolysis extent was much higher in boiled eggs (79%) than in poached and omelet ones (60 and 56%, respectively) under control GI conditions. Apparently, trypsin inhibitors present in white eggs seem to be inactivated as long as the food is exposed to 100 °C as well as a greater protein denaturation,^13,14^ leading to a greater extent in hard-boiled eggs than in poached and omelet eggs. It is well known that the different ways of egg cooking lead to different matrix structures, physical behavior, sensorial quality, and composition of eggs.^43^ Therefore, an impact of cooking eggs on digestibility was expected. In the case of omelet, the mixing and stirring of yolk with white egg seem to generate new protein–lipid organization that, together with the solid structure resulting from the heat treatment, would hinder the access of gastric and pancreatic proteases to the substrate and result in lower protein digestion.^23^ It is important to highlight that the extent of proteolysis achieved by the samples could be even higher than reported because the extent of proteolysis calculation has been just based on FAA without considering the possible short-chain peptides which are also bioabsorbable.

**Table 2:** Amino Acids Profile (g/100 g Initial Protein) Resulting from *In Vitro* Digestion of Hard-boiled, Poached and Omelet Eggs under Different Simulated GI Conditions (Control (C), Elderly 1 (E1), Elderly 2 (E2), Elderly 3 (E3) Models)^d^

	hard-boiled				poached				omelet			
amino acid	C	E1	E2	E3	C	E1	E2	E3	C	E1	E2	E3
alanine (Ala)	4.39 ± 0.15^cB^	3.68 ± 0.28^bC^	3.67 ± 0.37^bB^	2.50 ± 0.12^aB^	3.05 ± 0.42^aA^	3.21 ± 0.09^aB^	3.00 ± 0.34^aB^	2.63 ± 0.18^aB^	2.65 ± 0.34^bA^	2.23 ± 0.09^bA^	2.42 ± 0.13^bA^	1.76 ± 0.12^aA^
glycine (Gly)	1.48 ± 0.25^bB^	1.50 ± 0.24^bB^	1.53 ± 0.16^bC^	0.48 ± 0.12^aAB^	1.24 ± 0.26^bB^	1.16 ± 0.10^bB^	1.14 ± 0.04^bB^	0.51 ± 0.02^aB^	0.83 ± 0.12^bA^	0.66 ± 0.02^bA^	0.70 ± 0.08^bA^	0.33 ± 0.07^aA^
valine (Val)	5.77 ± 0.01^bB^	5.77 ± 0.64^bB^	6.07 ± 0.58^bC^	4.18 ± 0.34^aB^	4.78 ± 0.38^aA^	5.20 ± 0.09^aB^	4.52 ± 0.25^aB^	4.72 ± 0.38^aB^	4.49 ± 0.52^bA^	3.77 ± 0.10^abA^	3.94 ± 0.19^abA^	3.15 ± 0.49^aA^
leucine (Leu)	10.37 ± 0.26^bB^	10.08 ± 1.28^bB^	10.86 ± 1.08^bB^	7.49 ± 0.58^aAB^	8.72 ± 0.47^aA^	8.65 ± 0.26^aB^	7.88 ± 0.12^aA^	8.13 ± 0.46^aB^	8.91 ± 0.88^bA^	7.46 ± 0.37^aA^	7.60 ± 0.40^aA^	6.81 ± 0.30^aA^
isoleucine (Ile)	4.29 ± 0.07^bB^	4.24 ± 0.28^abB^	4.01 ± 0.92^abA^	3.12 ± 0.22^aAB^	3.63 ± 0.32^aA^	3.87 ± 0.12^aB^	3.10 ± 0.51^aA^	3.45 ± 0.40^aB^	3.51 ± 0.32^bA^	2.92 ± 0.17^aA^	3.01 ± 0.19^abA^	2.78 ± 0.18^aA^
threonine (Thr)	3.25 ± 0.26^bB^	2.96 ± 0.20^bC^	3.12 ± 0.34^bC^	1.77 ± 0.15^aB^	2.22 ±0.28^abA^	2.61 ± 0.06^cB^	2.19 ± 0.13^bcB^	1.88 ± 0.20^aB^	2.02 ± 0.33^bA^	1.79 ± 0.04^bA^	1.91 ± 0.05^bA^	1.39 ± 0.08^aA^
serine (Ser)	3.71 ± 0.18^bB^	3.43 ± 0.33^bB^	3.65 ± 0.38^bC^	1.40 ± 0.10^aB^	2.29 ± 0.21^abA^	2.96 ± 0.30^bB^	2.62 ± 0.19^abB^	1.87 ± 0.28^aC^	2.21 ± 0.39^bA^	1.97 ± 0.14^bA^	2.15 ± 0.12^bA^	1.13 ± 0.07^aA^
proline (Pro)	1.24 ± 0.05^bC^	1.15 ± 0.15^bB^	1.27 ± 0.16^bC^	0.67 ± 0.06^aB^	1.01 ± 0.10^abB^	1.15 ± 0.01^bB^	0.98 ± 0.11^abB^	0.76 ± 0.12^aC^	0.77 ± 0.06^bA^	0.67 ± 0.04^bA^	0.72 ± 0.05^bA^	0.46 ± 0.06^aA^
asparagine (Asn)	2.73 ± 0.56^aB^	2.48 ± 0.23^aB^	2.65 ± 0.28^aB^		2.00 ± 0.40^aAB^	2.74 ± 0.24^aB^	2.11 ± 0.38^aAB^		1.68 ± 0.22^bA^	1.42 ± 0.02^bA^	1.60 ± 0.19^bA^	0.40 ± 0.18^a^
aspartic acid (Asp)	2.40 ± 0.10^bB^	2.31 ± 0.32^bB^	2.32 ± 0.28^bB^	0.19 ± 0.06^aA^	1.83 ± 0.13^bA^	2.27 ± 0.08^bB^	2.10 ± 0.16^bB^	1.17 ± 0.26^aC^	1.55 ± 0.20^bA^	1.38 ± 0.09^bA^	1.38 ± 0.09^bA^	0.72 ± 0.03^aB^
methionine (Met)	3.24 ± 0.12^abB^	3.06 ± 0.53^abAB^	3.39 ± 0.39^bB^	2.57 ± 0.11^aA^	2.80 ± 0.24^aA^	3.00 ± 0.03^aB^	2.60 ± 0.10^aA^	2.58 ± 0.46^aA^	2.95 ± 0.27^bAB^	2.58 ± 0.17^abA^	2.56 ± 0.19^abA^	2.40 ± 0.17^aA^
glutamic acid (Glu)	3.20 ± 0.42^bB^	3.10 ± 0.18^abB^	3.12 ± 0.15^abB^	2.54 ± 0.18^aB^	3.02 ± 0.10^bB^	3.32 ± 0.10^cB^	2.97 ± 0.10^bB^	2.66 ± 0.07^aB^	2.20 ± 0.20^bA^	1.98 ± 0.05^bA^	1.99 ± 0.05^bA^	1.56 ± 0.05^aA^
phenylalanine (Phe)	6.56 ± 0.15^bB^	6.66 ± 1.10^bB^	7.35 ± 0.77^bB^	4.91 ± 0.27^aA^	5.90 ± 0.21^cA^	5.63 ± 0.30^bcAB^	4.84 ± 0.09^aA^	5.01 ± 0.44^abA^	6.27 ± 0.49^bAB^	5.18 ± 0.31^aA^	5.10 ± 0.27^aA^	4.64 ± 0.34^aA^
glutamine (Gln)	6.60 ± 0.11^bC^	5.85 ± 0.93^bC^	6.49 ± 0.77^bC^	4.20 ± 0.24^aB^	4.58 ± 0.41^aB^	4.48 ± 0.43^aB^	4.97 ± 0.84^aB^	4.22 ± 0.36^aB^	3.61 ± 0.22^cA^	3.42 ± 0.20^bcA^	3.10 ± 0.10^bA^	2.18 ± 0.12^aA^
lysine (Lys)	7.87 ± 1.18^bB^	7.36 ± 0.26^bB^	8.52 ± 0.31^bC^	4.33 ± 0.71^aB^	4.43 ± 0.43^aA^	6.37 ± 0.75^bB^	5.97 ± 0.21^bB^	5.17 ± 0.05^aC^	5.05 ± 0.84^bA^	4.36 ± 0.13^bA^	5.12 ± 0.60^bA^	3.08 ± 0.10^aA^
histidine (His)	2.32 ± 0.14^bB^	2.60 ± 0.43^bB^	2.85 ± 0.31^bC^	1.60 ± 0.09^aB^	2.19 ± 0.08^bB^	1.99 ± 0.20^abB^	2.03 ± 0.08^abB^	1.92 ± 0.11^aC^	1.76 ± 0.19^bA^	1.60 ± 0.07^abA^	1.71 ± 0.06^bA^	1.36 ± 0.07^aA^
tyrosine (Tyr)	6.96 ± 0.25^bC^	7.14 ± 0.72^bB^	7.62 ± 0.95^bC^	4.88 ± 0.16^aC^	3.48 ± 0.09^abA^	3.56 ± 0.06^abA^	3.12 ± 0.01^aA^	3.81 ± 0.41^bB^	4.77 ± 0.40^cB^	3.88 ± 0.22^bA^	3.64 ± 0.14^abB^	3.10 ± 0.25^aA^
tryptophan (Trp)	2.75 ± 0.06^abA^	3.01 ± 0.34^bcB^	3.53 ± 0.36^cB^	2.18 ± 0.12^aB^	2.65 ± 0.18^bA^	2.37 ± 0.27^abA^	2.18 ± 0.18^aA^	2.29 ± 0.06^abB^	2.66 ± 0.27^bA^	2.21 ± 0.17^aA^	2.18 ± 0.12^aA^	1.90 ± 0.14^aA^
cystine (Cys)									0.97 ± 0.26^a^	1.01 ± 0.02^a^	1.02 ± 0.12^a^	0.82 ± 0.05^a^

a,b,c Different lowercase letters indicate significant differences between models, with a significance level of 95% (*p* < 0.05).

A,B,C Different capital letters indicate significant differences between cooking methods, with a significance level of 95% (*p* < 0.05).

d Data shown are mean values from triplicates and the standard deviation.

Concerning the effect of GI alterations of elders on egg digestion, results also show that neither oral nor gastric alterations (E1 and E2) negatively impacted *in vitro* proteolysis extent (sum of the FAA released). Nevertheless, suboptimal intestinal conditions with reduced pancreatic and bile salts concentration coupled with an increase of residence time (E3) significantly reduced protein digestibility in both hard-boiled and omelet eggs. Proteolysis experimented reduction of 38 and 32% of the FAA released in hard-boiled and omelet eggs, respectively, under E3 GI conditions and compared to C. This result evidences the role of matrix organization, the proteins from solid matrices (hard-boiled and omelet) hinder to a greater extent than semiliquid matrices, the release and hydrolysis of proteins under suboptimal intestinal conditions.^44^ Poached egg resulted in a liquid yolk and semisolid white, which can be easily mixed with digestive fluids. In hard-boiled egg, both white and yolk acquired a solid structure, making the matrix degradation harder for its consequent hydrolyzation. In turn, omelet presents an emulsion-like structure of medium moisture in which protein network embeds lipid molecules and proteolysis has to occur before lipids can be made accessible to lipases.^45^ The intrinsic molecular properties of the egg proteins might determine enzyme accessibility, these properties being modified according to processing such as heat gelation. In fact, products with the same composition but different matrix structures can lead to different digestion patterns.^20^ In turn, Asensio-Grau et al.^23^ reported a higher impact of egg cooking methods on the digestibility of proteins, lipids, and xanthophylls bioaccessibility under exocrine pancreatic insufficiency (EPI) conditions than under healthy ones. Thus, poaching favored egg protein digestion under EPI conditions compared to other methods, mainly due to its semiliquid structure and lower degree of protein denaturation.

The essential amino acids (EAA)/nonessential amino acids (NEAA) ratio is also shown in Figure 2B. The EAA/NEAA ratio of cooked eggs digested under C model ranged from 1.78 to 2.14, this value being significantly lower in hard-boiled than in poached egg and omelet. A similar EAA/NEAA ratio was obtained from egg samples digested under E1 (oral alteration) and E2 (oral and gastric alterations) GI conditions. However, a considerable increase was found in samples digested mimicking the most suboptimal GI conditions given in elders (E3 model). According to this result, elderly intestinal conditions might favor the essential amino acids release to a greater extent than the nonessential ones, even if the total proteolysis extent was reduced under the E3 model. The predominant release of EAA than NEAA might be due to pancreatic enzymes specificity for certain peptide bonds,^46^ this effect being more relevant under a low enzymatic concentration (E3 model). The importance of EAA lies in muscle protein synthesis, as they are highly involved in this process.^47^ Therefore, even if the total FAA achieved under the most critical scenery resulted in reductions, this result would be especially relevant for elders suffering from sarcopenia, especially for the qualitative (referred to more EAA than NEAA) more than quantitative (total FAA extent) protein consumption point of view.

Complementarily, Figure 2 shows the amino acidic contents (g amino acids/100 g of initial protein) of hydrophobic amino acids (HAA), positively charged amino acids (PCAA), negatively charged amino acids (NCAA), aromatic amino acids (AAA), and sulfur-containing amino acids (SCAA). The presence of HAA and PCAA in the samples, especially Tyr, Met, His, and Lys, has been found to improve the antioxidant properties of peptides. In turn, amino acids with a large side group such as tryptophan (AAA with an indolic group) and histidine (PCAA with an imidazole group) contribute to the antioxidant potential of peptides but in the case as hydrogen donors. Additionally, peptide–lipid interactions can promote, or even improve, the antioxidant effects of peptides as a consequence of their hydrophobic properties.^46^ Moreover, some of the PCAAs are involved in upregulation of genes involved in mitochondrial biogenesis, offering another mechanism for increased oxidation of long-chain fatty acids and glucose in insulin-sensitive tissues.^48^ Likewise, methionine (with an SCAA character) besides histidine, serine, and glycine are the major donors of 1-carbon groups.^48^ In fact, diet supplementation with some NCAA, PCAA, and SCAA (e.g., glutamine, arginine, and N-acetyl-cysteine, respectively) are proposed for contributing to oxidative defense and immune function.^48^ After digestion under C conditions, the higher presence of amino acids with hydrophobic character (HAA) (sum of alanine, valine, isoleucine, leucine, tyrosine, phenylalanine, tryptophan, proline, methionine, and cysteine) in the amino acid profile (between 36 and 45.6 g HAA/100 g protein) is notable, corresponding to hard-boiled the greatest content compared to the other chemical groups. HAA content experimented, however, a notable decrease under E3 GI conditions in hard-boiled and omelet. On the contrary, sulfur-containing amino acid (SCAA) (sum of cysteine and methionine) was the least present chemical group (between 3.2 and 3.9 g/100 g protein under C model), regardless of the cooking methods or GI conditions. Slight reductions in SCAA content in the three cooking methods were shown, but only a statistical effect of elderly GI conditions was found in poached and omelet. Regarding the positively (PCAA) and negatively (NCAA) charged as well as the aromatic (AAA) amino acid contents, values obtained under E3 model were significantly lower than those found in the amino acid profile under C model in hard-boiled eggs and omelet. However, the hard-boiled egg seems to provide greater amounts of almost all of the chemical groups (excepting of SCAA) and also was the most affected sample by elderly alterations, with reductions up to 53% for NCAA under E3 GI conditions.

Besides the nutritional point of view, protein hydrolysates exert a positive impact on human health such as radical scavenging and reducing potential when large amounts of hydrophobic sulfur-containing amino acids such as cysteine, histidine, tryptophan, tyrosine, and phenylalanine are released.^32,49^ The contribution of scavenging free radicals to human health promotion has been stated as delayers of associated oxidative damage to the physiological macromolecules. They play, therefore, a crucial role against cardiovascular, inflammatory, and aging-induced degenerative diseases as well as cancers.^50^

### Effect of Egg Cooking on Lipid Digestibility in Elders

The molar percentages of acyl groups (AG) of the products derived from triglyceride hydrolysis (TG) after digestion are presented in Table 3. As expected, 90% of the total fat in cooked eggs was present as TG before digestion. After GI digestion under C conditions, lipolysis extent achieves values of 99.7, 95.6, and 94.9% for hard-boiled eggs, poached eggs, and omelet, respectively. The conversion due to the hydrolytic action of pancreatic lipase of TG was mainly into FFA with values of 77.23, 80.92, and 71.18% in hard-boiled eggs, poached eggs, and omelet, respectively; followed by 1,2-DG, 2-MG, 1-MG, and 1,3-DG. In omelet samples, fat globules could be trapped in a well-stable protein network resulting from mixing and the posterior thermal treatment. Thus, the protein enzymatic breakdown occurs before lipids can be made accessible to lipases.^45^ These lipid–protein interactions slow down the accessibility of enzymes to the substrate, leading to lower conversion of TG into FFA together with lower matrix degradation compared to other methods.^23^

**Table 3:** Molar Percentages of Acyl Groups (AG) Supported on the Different Glyceryl Backbone Structures (TG, 1,2-DG, 1,3-DG, 2-MG, 1-MG) and Free Fatty Acids (FFA) and Cholesterol Content (mg/g Fat), Present in Non-digested (ND) and Digested Hard-boiled, Poached and Omelet Eggs; *In Vitro* GI Models: Control (C), Elderly 1 (E1), Elderly 2 (E2), Elderly 3 (E3)^e^

cooking method	GI conditions	AG_Tg_(%)	AG1,2DG(%)	AG_1,3-DG_ (%)	AG2-MG(%)	AG1-MG(%)	FFA (%)	absorbable fraction (%)^f^	non-absorbable fraction (%)^g^	lipolysis extent (%)^h^	cholesterol (mg/g fat)
hard-boiled	ND	89.57 ± 2.10				10.42 ± 2.08		10.43 ± 2.10		10.43 ± 2.10	54.14 ± 2.92^bA^
	C	0.32 ± 0.26^aA^	12.84 ± 0.44^cA^	1.021 ± 0.002^cB^	5.61 ± 0.18^bB^	3.00 ± 0.31^aB^	77.23 ± 0.06^aB^	85.83 ± 0.18^aC^	13.86 ± 0.44^cB^	99.68 ± 0.27^bC^	50.91 ± 4.91^abAB^
	E1	0.30 ± 0.07^aA^	11.81 ± 0.06^bA^	0.84 ± 0.09^bB^	5.70 ± 0.10^bB^	2.87 ± 0.20^aB^	78.97 ± 1.04^aB^	87.55 ± 0.74^aC^	12.66 ± 0.03^bA^	100.20 ± 0.78^bC^	47.82 ± 4.22^abA^
	E2	0.96 ± 0.22^aA^	11.88 ± 0.23^bcB^	0.53 ± 0.06^aB^	6.15 ± 0.27^bB^	3.12 ± 0.02^aB^	78.09 ± 1.16^aB^	87.36 ± 1.41^aB^	12.41 ± 0.17^bA^	99.77 ± 1.24^bB^	46.65 ± 7.18^abA^
	E3	4.73 ± 2.69^bAB^	6.53 ± 0.49^aA^	2.63 ± 0.04^dB^	1.35 ± 0.21^aA^	3.68 ± 0.05^bB^	81.09 ± 3.41^aB^	86.12 ± 3.14^aAB^	9.15 ± 0.45^aA^	95.27 ± 2.69^aAB^	51.19 ± 5.68^aA^
poached	ND	89.98 ± 0.29				9.99 ± 0.30		10.02 ± 0.29		10.02 ± 0.29	56.39 ± 2.81^aA^
	C	4.41 ± 0.42^bB^	11.16 ± 0.44^bcB^	0.91 ± 0.13^aB^	1.82 ± 0.02^bA^	0.79 ± 0.07^bA^	80.92 ± 1.09^aC^	83.53 ± 0.99^aB^	12.06 ± 0.57^bcA^	95.59 ± 0.42^aB^	60.31 ± 4.06^aB^
	E1	2.40 ± 0.19^aB^	12.46 ± 0.27^cB^	1.00 ± 0.03^aC^	1.46 ± 0.06^aA^	0.49 ± 0.09^aA^	82.19 ± 0.20^aC^	84.14 ± 0.05^aB^	13.46 ± 0.24^cB^	97.60 ± 0.19^bB^	63.08 ± 4.48^aB^
	E2	2.01 ± 0.88^aAB^	10.83 ± 0.86^bA^	0.81 ± 0.07^aC^	1.33 ± 0.20^aA^	0.46 ± 0.04^aA^	84.56 ± 0.17^bC^	86.34 ± 0.06^bB^	11.65 ± 0.94^bA^	97.99 ± 0.88^bAB^	58.69 ± 8.46^aA^
	E3	2.32 ± 0.39^aA^	8.99 ± 0.04^aB^	0.82 ± 0.02^aA^	1.78 ± 0.04^bB^	0.61 ± 0.07^abA^	85.47 ± 0.48^bC^	87.86 ± 0.37^cB^	9.81 ± 0.02^aA^	97.68 ± 0.39^bB^	56.65 ± 7.39^aA^
omelet	ND	90.86± 1.01				9.63 ± 1.09		9.64 ± 1.11		9.64 ± 1.11	52.27 ± 1.58^bA^
	C	5.11 ± 0.24^abB^	13.06 ± 0.02^abA^	0.30 ± 0.10^aA^	7.22 ± 0.13^cC^	3.11 ± 0.59^aB^	71.18 ± 0.09^aA^	81.51 ± 0.37^abA^	13.37 ± 0.13^aB^	94.89 ± 0.24^aA^	45.29 ± 0.86^abA^
	E1	5.49 ± 0.08^bC^	13.61 ± 0.38^bC^	0.68 ± 0.02^abA^	6.22 ± 0.07^bC^	3.10 ± 0.04^aB^	71.67 ± 0.50^abA^	80.98 ± 0.61^aA^	14.29 ± 0.39^aC^	95.28 ± 1.00^aA^	47.33 ± 1.72^abA^
	E2	3.90 ± 1.10^aB^	11.97 ± 0.37^aB^	0.29 ± 0.12^aA^	7.03 ± 0.28^cC^	3.64 ± 0.12^aC^	73.21 ± 1.02^abA^	83.87 ± 0.62^bA^	12.26 ± 0.49^aA^	96.12 ± 1.10^aA^	49.83 ± 5.58^abA^
	E3	4.28 ± 0.09^abB^	13.03 ± 1.02^abC^	0.93 ± 0.39^bA^	4.11 ± 0.38^aC^	3.38 ± 0.61^aB^	74.27 ± 1.74^bA^	81.76 ± 1.50^abA^	13.96 ± 1.41^aB^	95.72 ± 0.09^aA^	47.18 ± 2.25^aA^

a,b,c,d Different lowercase letters indicate significant differences between models, with a significance level of 95% (*p* < 0.05).

A,B,C Different capital letters indicate significant differences between cooking methods, with a significance level of 95% (*p* < 0.05).

e Data shown are mean values from triplicates and the standard deviation.

f Absorbable fraction includes AG_2-MG_% + AG_1-MG_% + FFA%.

g Nonabsorbable fraction AG_1,2-DG_% + AG_1,3-DG_%.

h Lipolysis extent represent the summarize.

Regarding the elderly GI conditions and their effect on lipid digestion, oral, gastric, and intestinal alterations negatively impact the absorbable fraction of hard-boiled, poached, and omelet eggs. In fact, a significant increase (*p* < 0.05) was noted in E3 with respect to C in poached egg. Nevertheless, the nonabsorbable fraction was slightly, but significantly, reduced in hard-boiled and poached eggs, and therefore the total lipolysis extent. Therefore, a longer intestinal transit time would be responsible for exerting a positive effect on lipid digestion,^51^ even under reduced pancreatic lipase and bile concentrations (E3 model).

Finally, the cholesterol contents (Table 3) of hard-boiled, poached, and omelet eggs before digestion were similar. These results are in agreement with those reported by Hur et al.,^52^ where the cholesterol content in pork patties was not affected by different cooking methods. However, cholesterol stability was slightly reduced in hard-boiled and omelet eggs after *in vitro* digestion. The decrease of cholesterol could be attributed to the higher formation of cholesterol oxidation products during *in vitro* digestion,^53^ being both physicochemical and enzymatic conditions the oxidation promoters.^54^ Also, microwave cooking^52^ might be co-responsible for the higher oxidative damage of cholesterol during the posterior GI digestion.

### Vitamins A and D3 Bioaccessibility in Eggs: Impact of Cooking and GI Alterations in Elders

**Figure 3:**
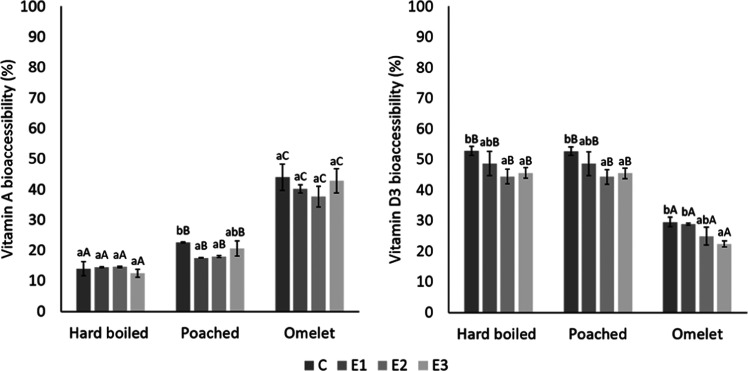
Vitamin A and D3 bioaccessibility achieved in hard-boiled, poached, and omelet eggs *in vitro* digested under different GI conditions (control (C), Elderly 1 (E1), Elderly 2 (E2), and Elderly 3 (E3) models). Different lowercase letters indicate significant differences between models, and different capital letters indicate significant differences between cooking methods, with a significance level of 95% (*p* < 0.05).

Figure 3 shows the vitamin A and D3 bioaccessibility (%) of hard-boiled, poached, and omelet eggs. Similarly to macronutrient digestibility, the structure matrix seems to be responsible, to a certain extent, for to the differences found in terms of solubilization and micellar incorporation of the micronutrients. Hence, it was found that the higher the complexity of structured food matrices (i.e., omelet), the minor the fat-soluble vitamin bioaccessibility present in the yolk.^23,55^ Vitamin D3 bioaccessibility values under standardized GI conditions (C) agree with this behavior. Nevertheless, vitamin A bioaccessibility was higher in omelet than in hard-boiled or poached eggs. Vitamin A has been reported to experiment oxidation along digestion, leading to a reduced final concentration but increasing the presence of other compounds such as β-ionone, 2,2,6-trimethylcyclohexanone,β-cyclocitral,(E)-5,6-epoxy-β-ionone, ionene, β-homocyclocytral, and dihydroactinidiolide.^56^ Hence, omelet structure could exert a protective effect on vitamin A against oxidation reactions and explain a higher vitamin A bioaccessibility in omelet than in hard-boiled and poached eggs.

With respect to vitamin bioaccessibility under GI conditions of elders (E1, E2, and E3), vitamin D3 release from all egg products was significantly reduced under E3 model conditions. However, no statistically significant differences were found in vitamin A bioaccessibility values achieved under C and E3 digestion conditions. Only vitamin A release from poached eggs seems to be negatively affected when oral and gastric conditions were suboptimal as in E1 and E2 simulations.

Liposoluble compounds release is dependent on their solubilization favored by bile acids presence. Thus, it was expected to obtain lower bioaccessibility values of both vitamins under reduced bile salts concentration occurring in the E3 model. Nevertheless, only vitamin D3 was affected by this suboptimal intestinal condition.^57^

### Descriptive Relationship Among Digestibility, Egg Cooking Methods, and Elderly GI Conditions

**Figure 4:**
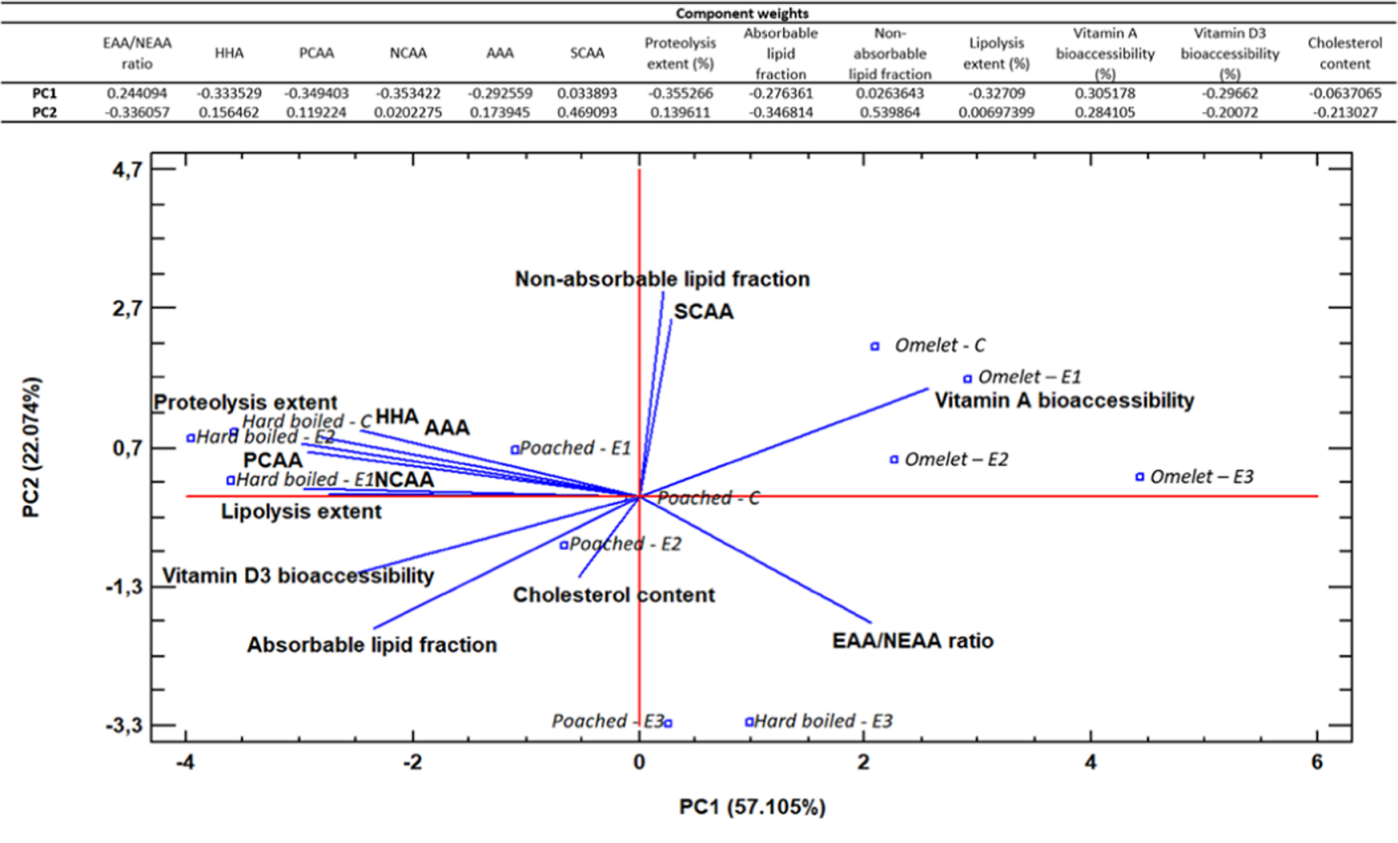
Biplot and component weights of the different end-digestion products of proteins (proteolysis extent, EAA/NEAA ratio, HAA, PCAA, NCAA, AAA, and SCAA contents), lipids (cholesterol content, absorbable, nonabsorbable, and total lipolysis extents), and micronutrients (vitamin A and D3 bioaccessibility) and their association with the binomial cooked eggs (hard-boiled, poached, and omelet) under GI conditions (Control (C), Elderly 1 (E1), Elderly 2 (E2), Elderly 3 (E3)) obtained by means of a principal components analysis (PCA).

A PCA was performed to assess the relationship between digestion end products from a descriptive point of view (Figure 4). Also, the component weights and the scores of hard-boiled, poached, and omelet eggs digested under the simulated GI conditions (C, E1, E2, and E3) are included. The first two principal components of the analysis explain 79.179% of the total variance of the digestibility in the samples (PC1: 57.105% and PC2: 22.074%). Using the number of factor loads for two main components, it was identified which variables significantly affect the components C1 and C2. Vitamin bioaccessibility, lipolysis extent, as well as the HHA, PCAA, NCAA, and total (sum of the FAA released) proteolysis extents have the most significant impact on the value of the PC1. On the other hand, absorbable and nonabsorbable lipid fractions, SCAA, and EAA/NEAA ratio presented the most significant impact on the PC2 value. As a result, this procedure allows the analysis of the two-dimensional space that was created based on the main components. In the score plot, the proximity between samples indicates similar behavior in terms of digestibility. In PC1, it is noted that omelet, located at the upper right side of the plot, exhibits a digestion pattern different from those of hard-boiled and poached eggs, located at the left side of the plot. PC2 seems to distinguish vitamin A bioaccessibility (higher in omelet) and samples with a higher EAA/NEAA ratio after digestion. Overall, PCA shows the narrow relationship between: proteolysis and lipolysis extents; the amino acids chemical classifications (excepting SCAA) with the proteolysis extent and the vitamin D3 bioaccessibility; and the absorbable lipid fraction and the cholesterol content with the lipolysis extent.

In sum, GI alterations appearing with aging negatively affect the ovo-protein digestibility with a reduction of up to 37% in the FAA released, compared with total FAA extents obtained under control conditions. Hard-boiled or poached method was more advisable than omelet preparation to maximize the proteolysis extent (sum of FAA released) under elderly conditions. A notable increase in the release of essential amino acids, compared with the nonessential ones, was also noted under simulated elderly GI conditions. Neither total lipolysis extent nor lipidic absorbable fraction is compromised with aging. Nevertheless, omelet preparation plays a significant role against the absorbable lipid fraction, mainly in free fatty acid release. Finally, vitamin D3, lipolysis, and proteolysis extents seem to be positively linked, especially in hard-boiled and poached eggs under elderly GI conditions. It could be stated that poached and omelet preparations might be more advisable than hard-boiled in terms of net supply of bioaccessible vitamin A for elders, while the bioaccessible vitamin D3 contents provided are very similar regardless of the cooking method. Therefore, this study provides a better understanding of egg protein and lipid hydrolysis, together with liposoluble vitamin bioaccessibility, under GI conditions of elderlies and as a function of cooking method. This information tries to contribute to establishing accurate dietary recommendations addressed to this population group.

## Abbreviations

**GI**: gastrointestinal
**C**: standardized gastrointestinal condition
**E1**: elderly oral alteration
**E2**: elderly oral and gastric alterations
**E3**: elderly oral, gastric, and intestinal alterations
**DIAAS**: digestible indispensable amino acid score
**U**: enzymatic units
**HPLC**: high-performance liquid chromatography
**SGF**: simulated gastric fluid
**SIF**: simulated intestinal fluid
**^1^H NMR**: proton nuclear magnetic resonance
**FAA**: free amino acids
**EAA**: essential amino acids
**NEAA**: nonessential amino acids
**HAA**: hydrophobic amino acids
**PCAA**: positively charged amino acids
**NCAA**: negatively charged amino acids
**AAA**: aromatic amino acids
**SCAA**: sulfur-containing amino acids
**GC–MS**: gas chromatography–mass spectrometry
**AG**: acyl groups
**MG**: monoglycerides
**DG**: diglycerides
**TG**: triglycerides
**FFA**: free fatty acids
**ANOVA**: analysis of variance
**LSD**: less significant difference
**PC**: principal component
**PCA**: principal component analysis
**Ala**: alanine
**Gly**: glycine
**Val**: valine
**Leu**: leucine
**Ile**: isoleucine
**Thr**: threonine
**Ser**: serine
**Pro**: proline
**Asn**: asparagine
**Asp**: aspartic acid
**Met**: methionine
**Glu**: glutamic acid
**Phe**: phenylalanine
**Gln**: glutamine
**Lys**: lysine
**His**: histidine
**Tyr**: tyrosine
**Trp**: tryptophan
**Cys**: cystine
**ND**: nondigested
